# De Novo Proliferative Glomerulonephritis With Monoclonal Immunoglobulin Deposits (PGNMID) in a Renal Transplant Recipient

**DOI:** 10.7759/cureus.111522

**Published:** 2026-06-25

**Authors:** Ram Prabahar Murugesan, Sathiyan Sivanandam, Jayanivash Jayam, Anila A Kurian

**Affiliations:** 1 Department of Nephrology, SRM Institute of Medical Sciences (SIMS) Hospital, Chennai, IND; 2 Renopathology, Renopath Center for Renal and Urological Pathology Pvt. Ltd., Chennai, IND

**Keywords:** allograft dysfunction, de novo glomerular disease, mgrs, monoclonal gammopathy, pgnmid

## Abstract

Proliferative glomerulonephritis with monoclonal immunoglobulin deposits (PGNMID) represents a distinct glomerular pathology, classified under monoclonal gammopathy of renal significance (MGRS). In renal transplant, PGNMID usually develops as a recurrent disease, but can rarely arise de novo. Recurrence is relatively common, typically appearing within five to six months post-transplant, and is linked to poor graft outcomes. De novo PGNMID is exceedingly rare, with few reported in the literature. It generally presents in the late post-transplant period, with a more indolent clinical course and a variable response to immunotherapy. This case report is of a 50-year-old patient who had diabetic nephropathy as his native kidney disease. This report documents a unique instance of de novo PGNMID, occurring three years post-transplantation with persistent allograft dysfunction. The transplant kidney biopsy showed mesangial hypercellularity with immunoglobulin (Ig)G and kappa light chain deposition by immunofluorescence; however, electron microscopy was non-contributory due to the absence of viable glomeruli. Despite extensive evaluation, we could not identify any clone contributing to the MGRS in the bone marrow, nor could we identify any other evidence of lymphoproliferative disease on positron emission tomography-computed tomography (PET-CT). We managed the patient with empirical clone-directed therapy against a likely B-cell clone using Rituximab. During rituximab therapy, the patient developed E. coli urosepsis, which was managed successfully. At the last follow-up, graft function remained stable without progression, although the duration of follow-up is limited.

## Introduction

Proliferative glomerulonephritis with monoclonal immunoglobulin deposits (PGNMID) is a rare glomerular injury, accounting for 0.17-0.27% of all renal biopsies [1,2]. PGNMID is a type of kidney injury caused by the abnormal deposition of monoclonal antibodies inside the glomeruli. The hallmark of PGNMID is "light-chain restriction," meaning the biopsy shows staining for only one specific type of light chain (Kappa or Lambda). In the post-transplant period, PGNMID may recur or develop de novo. Recurrent PGNMID has a median onset of five to six months and carries a poor prognosis [3]. De novo presentation remains rare. Since the first case series was reported, the pathogenesis of PGNMID has become better understood and is now recognized as part of the spectrum of monoclonal gammopathy of renal significance (MGRS).

MGRS denotes a group of kidney diseases caused by a toxic monoclonal immunoglobulin produced by a small, slow-growing, indolent clone of B-cells or plasma cells that do not meet the standard criteria for overt myeloma. However, this monoclonal protein can cause severe, progressive kidney damage. Identifying the clone as a plasma cell or B cell clone helps in providing appropriate clone-directed therapy for better clinical outcomes. But efforts to identify the monoclonal protein in urine or serum are fruitful in only one-third of patients [1], and identification in de novo PGNMID is even lower. It has also been reported to occur in the presence of infections (Parvovirus B19, Hepatitis C virus (HCV), Epstein-Barr virus (EBV)) and malignancies other than myeloma, like chronic lymphocytic leukemia (CLL) or post-transplant lymphoproliferative disease (PTLD) [2]. PTLD represents a spectrum of diseases, ranging from abnormal lymphoid hyperplasia to aggressive forms of lymphoma, and is often associated with EBV infections. In recurrent PGNMID post-transplant, most cases progress to graft failure [3], whereas de novo PGNMID shows a variable response to immunotherapy. A few case reports describe improvement in renal function following management of underlying infections such as EBV/BK virus in de novo PGNMID [4]. Identification of the clone responsible for the renal lesion is very important in the management of MGRS [1].

## Case presentation

Native kidney disease

**Figure 1:**
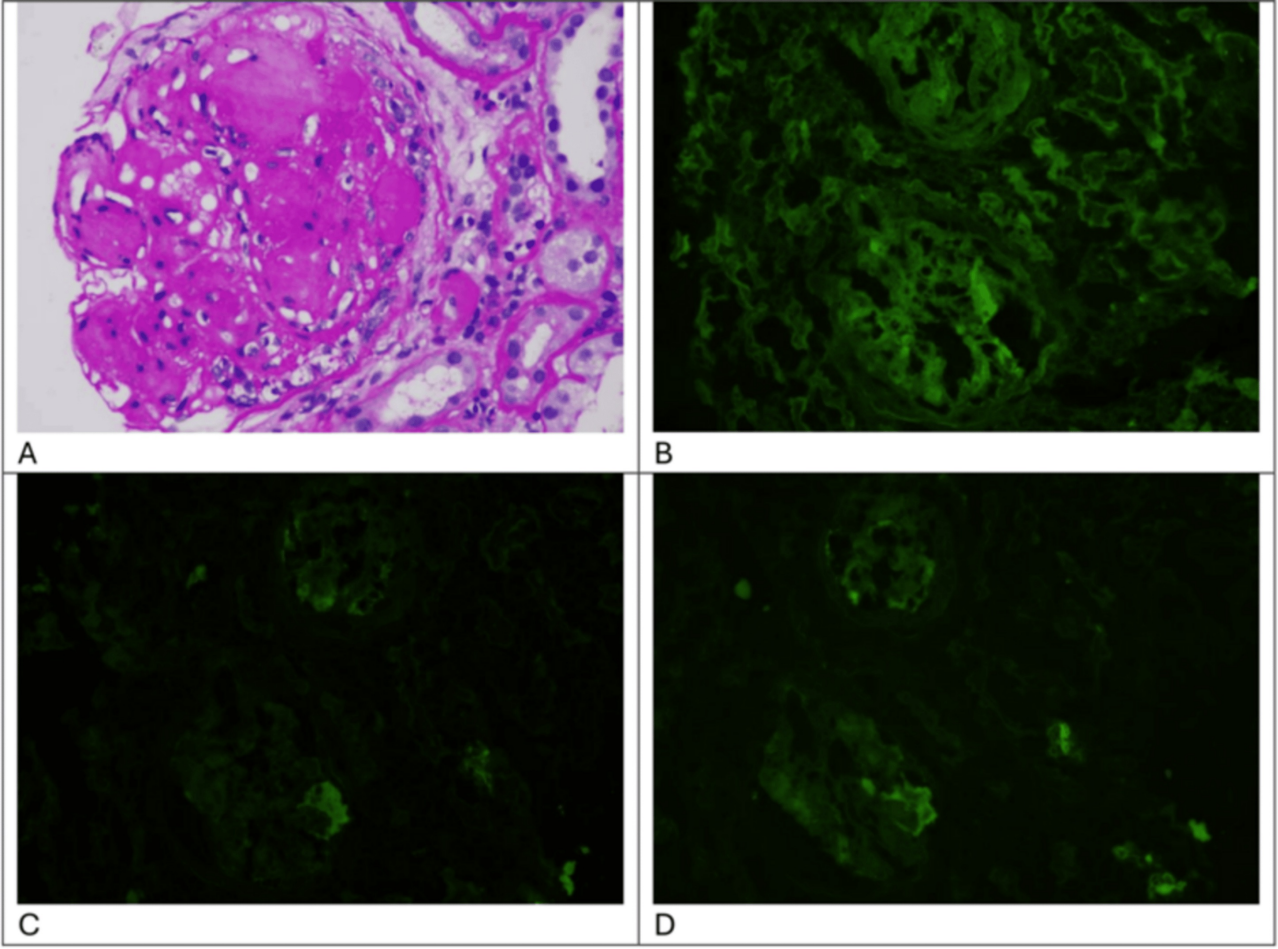
Light microscopy and immunofluorescence renal biopsy images Light microscopy and immunofluorescence findings from the renal biopsy showed diabetes mellitus nephropathy in the native kidney. A - PAS Stain (x40) showing diffuse and global glomerulosclerosis, B - IGG (++) in immunofluorescence microscopy, C - kappa light chain staining negative, D - Lambda light chain staining negative.

A 50-year-old male with diabetes for a duration of seven years, hypertension for three years, and hypothyroidism presented with oliguria and edema in 2017. He had no previous history of kidney disease. Evaluation revealed massive proteinuria and severe renal failure. Serum creatinine at presentation was 4.3 mg/dl, and 24-hour urine protein was 8.3 g, and there was no microscopic hematuria. Serum immunofixation electrophoresis did not show any monoclonal immunoglobulin. We did a renal biopsy, which showed nodular and diffuse glomerulosclerosis consistent with diabetic kidney disease (Figure 1). The initial renal biopsy showed no evidence of monoclonal light chain deposition, and we noted no cellular proliferation. We therefore managed him with optimal medical therapy, which included angiotensin receptor inhibitors, optimal control of hypertension (HT) (blood pressure (BP) < 130/80 mmHg), and glycemic status. He progressed gradually to end-stage renal disease in 2019 and was on maintenance hemodialysis for two years. He maintained a hemoglobin level of around 10-12 g/dl in dialysis.

Renal transplantation and the early post-transplant period

In November 2021, the patient underwent living donor ABO-compatible renal transplantation from his brother, aged 40 years, who was a haplomatched donor. The patient received induction immunosuppression with thymoglobulin and intravenous methylprednisolone, and he was on triple immunosuppression with tacrolimus, mycophenolate mofetil, and prednisolone. In the immediate post-transplant period, the patient developed allograft dysfunction. Serum creatinine was 2.3 mg/dl on post-operative day 5. Tacrolimus trough levels were 8.3 ng/ml. Renal biopsy revealed acute tubular injury with no evidence of rejection. IgG, IgM, IgA, and C3 were negative. Kappa and lambda light chains were negative. C4d staining was also negative. We suspected that the cause of the acute tubular injury was transient hypotension during surgery. Subsequently, renal function improved, and he maintained normal graft function with serum creatinine of 0.96 mg/dl after two weeks. 

Late post-transplant period

By September 2024, three years following renal transplant, he developed progressive graft dysfunction (serum creatinine 2.6 mg/dl, ++ albuminuria, microscopic haematuria, urine protein-creatinine ratio 3.8) (Table 1). A Doppler ultrasound of the transplant kidney was normal. Tacrolimus trough level was 7.5 ng/dl. Serum cytomegalovirus (CMV) and BK virus PCRs were negative.

**Table 1:** Laboratory values *The normal serum free kappa/lambda light chain ratio is 0.26-1.6. In the presence of renal impairment, the serum free kappa/lambda ratio is 0.37-3.1 if eGFR <60mL/min/1.73 m^2^. eGFR: Estimated Glomerular Filtration Rate, CKD-EPI: Chronic Kidney Disease Epidemiology Collaboration.

Laboratory parameters with date	Patient values	Normal reference range
Baseline serum creatinine (November 2021)	0.96 mg /dL	0.8 - 1.17 mg/dL
eGFR by CKD-EPI equation (November 2021)	98 ml/min/1.73m^2^	>60 ml/min/1.73m^2^
Serum creatinine (September 2024)	2.6 mg /dL	0.8 - 1.17 mg/dL
eGFR by CKD-EPI equation (September 2024)	29 ml/min/1.73m^2^	>60 ml/min/1.73m^2^
Urine spot protein-creatinine ratio (September 2024)	3.8 mg/mg creatinine	less than 0.20 mg/mg creatinine
24-hour urine protein (September 2024)	4306mg/day	less than 150 mg/day
Tacrolimus trough levels (September 2024)	7.5 ng/ml	5-8 ng/ml
Urine Bence Jones protein (September 2024)	negative	negative
Serum M protein immunofixation electrophoresis (September 2024)	negative	negative
Serum kappa free light chain(September 2024)	136 mg/L	3.3 - 19.4 mg/L
Serum lambda free light chain (September 2024)	102 mg/L	5.7 - 26.3 mg / L
Serum kappa/lambda free light chain ratio (September 2024)*	1.33	0.37 - 3.1 if eGFR less than 60ml/min/1.73m^2^
Serum calcium (September 2024)	8.3 mg/dL	8.5 - 10.2 mg/dL

**Figure 2:**
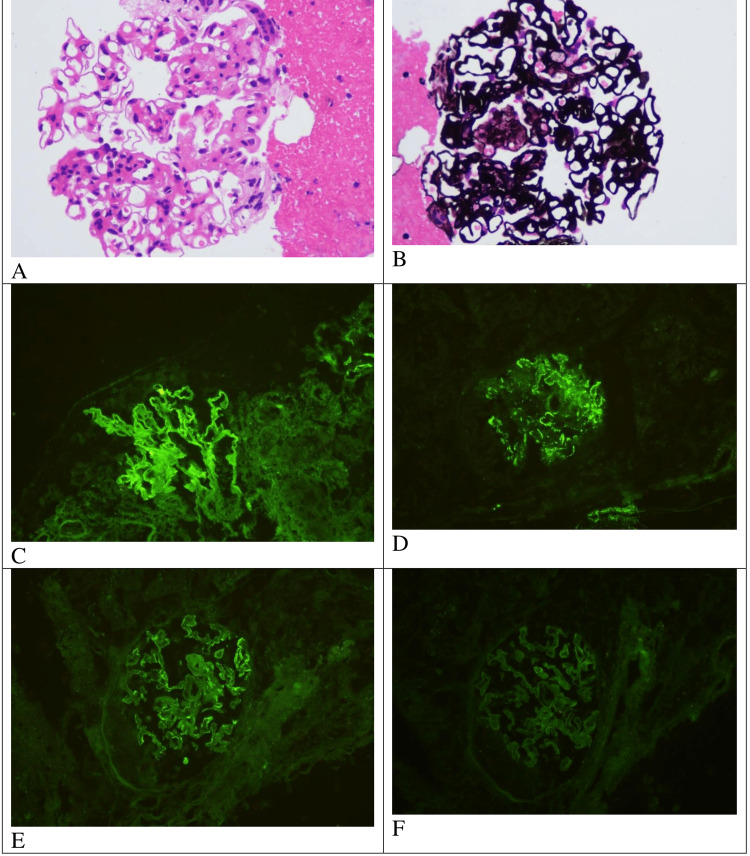
Light microscopy and immunofluorescence renal biopsy images showing PGNMID with kappa monoclonal immune deposition PGNMID: Proliferative glomerulonephritis with monoclonal immunoglobulin deposits. (A) H&E stain (40X) showing mesangial and endocapillary hypercellularity, (B) Periodic acid–Schiff (PAS) stain (40X) showing segmental double contouring of glomerular basement membrane, (C) IGG (+++), D-C3 (++), E-Kappa light chain positive, F-lambda light chain negative.

Therefore, we did a renal biopsy, and light microscopy revealed mesangial and segmental hypercellularity in 6/7 glomeruli, and segmental double-contouring in 40% of capillary loops. The Congo red stain for amyloid was negative. Immunofluorescence (IF) microscopy demonstrated strong IgG (+++) and C3 (++) granular positivity in glomerular capillary loops and selective kappa light-chain positivity (Figure 2). Lambda light chain staining was negative. C4d immunostaining was negative. An electron microscopy sample revealed no viable glomeruli for analysis. However, the tubulointerstitium showed no electron-dense deposits or viral particles. 

Serum immunofixation electrophoresis did not show a monoclonal M protein. Urine monoclonal protein evaluation and urine Bence Jones protein were negative. Serum kappa and lambda free light chain assays were 136 mg/L and 102 mg/L, respectively. The kappa/lambda free light chain ratio in serum was 1.33. Serum kappa/lambda ratio of 1.33 is normal for this eGFR level (reference: 0.37-3.1 if eGFR <60 mL/min/1.73m²). Urine protein electrophoresis and immunofixation did not show any monoclonal protein. Urine Bence-Jones protein test was negative. Serum corrected calcium was 8.8 mg/dl. Bone marrow biopsy was negative for plasma cell dyscrasias. Flow cytometry of the bone marrow showed 3% plasma cells with no surface light chain restriction, and there was no clonal proliferation of cells.

Positron emission tomography-computed tomography (PET-CT) did not reveal any skeletal osteolytic lesions; no evidence of post-transplant lymphoproliferative disease (PTLD), chronic lymphocytic leukemia (CLL), or other malignancies. In view of reported incidences of PGNMID associated with infections, we conducted testing for HCV RNA PCR, HBV DNA, EBV DNA, Parvovirus B19, CMV PCR, and BK Virus PCR, all of which were negative. Serum cryoglobulin and serum RA factors were also negative.

We established a diagnosis of PGNMID based on renal biopsy. Since the patient had renal dysfunction, microscopic haematuria, and nephrotic-range proteinuria, we treated the patient with empirical clone-directed therapy against a B-cell clone. The patient received six fortnightly doses of rituximab (500 mg). He continued receiving tacrolimus and mycophenolate mofetil at previous doses. During this period, he developed E. coli urosepsis, which we treated successfully. His serum creatinine remained stable (2.67 mg/dl after completing rituximab therapy). At last, at follow-up in January 2026, his serum creatinine was 2.6 mg/dL, serum kappa/lambda free light chain ratio was 1.30 (serum kappa free light chain 146 mg/L, serum lambda free light chain 112 mg/L). There was no progression of allograft dysfunction (Table 2). 

**Table 2:** Laboratory values pre- and post-rituximab therapy eGFR: Estimated Glomerular Filtration Rate, CKD-EPI: Chronic Kidney Disease Epidemiology Collaboration, PGNMID: Proliferative glomerulonephritis with monoclonal immunoglobulin deposits.

Date post-renal transplant	September 2024 (Pre rituximab therapy, at diagnosis of PGNMID)	January 2025 (post rituximab therapy)	February 2026(at last follow up)	Normal reference range
Serum creatinine	2.6 mg/dL	2.67 mg/dL	2.6 mg/dL	0.95 - 1.17 mg/dL
eGFR by CKD-EPI equation	29 ml/min/1.73m^2^	28 ml/min/1.73m^2 ^	29 ml/min/1.73m^2^	> 60 ml/min/1.73m^2^
Urine spot protein creatinine ratio	3.8 mg/mg creatinine	3.5 mg/mg creatinine	3.6 mg/mg creatinine	< 0.15 mg/mg creatinine
Serum albumin	3.5 g/dL	3.3 g/dL	3.4 g/dL	3.5 - 5 g/dL

A key limitation of this case is the absence of electron microscopy (EM) confirmation of the diagnosis. The biopsy sample contained no viable glomeruli for EM analysis. While the combination of light microscopy (mesangial hypercellularity, double contouring) and immunofluorescence (IgG3+, C3+, kappa-restricted) is highly suggestive of PGNMID, definitive diagnosis according to most classification systems requires EM demonstration of characteristic deposits. We acknowledge this limitation.

## Discussion

At present, the literature has reported only a handful of cases of de novo PGNMID (Table 2), whereas recurrent PGNMID is much more common. This patient’s native kidney disease was biopsy-proven diabetic nephropathy. Following transplantation, the immediate biopsy showed only acute tubular injury. However, three years later, he had new-onset graft dysfunction, which prompted a second biopsy that demonstrated PGNMID. Systemic evaluation failed to detect circulating monoclonal protein or evidence of myeloma or other hematological malignancies, findings that are consistent with other anecdotal cases (5-9). The low detection of monoclonal protein in PGNMID may be due to it being at such a low level that it cannot be detected by currently available tests. Detectable monoclonal protein in recurrent PGNMID is observed in about 20% of patients only, despite a high recurrence rate of 89% [3]. Among reported patients with de novo PGNMID, the identification of monoclonal protein is even rarer. Immunosuppressive therapy may partially suppress systemic monoclonal protein expression, explaining their negativity in such cases. 

**Table 3:** Reported cases of Denovo PGNMID SPK: Simultaneous Pancreas Kidney transplantation, CIN: Chronic Interstitial Nephritis, FSGS: Focal Segmental Glomerulosclerosis, PKD: Polycystic Kidney Disease, MPGN: Membrano Proliferative Glomerulonephritis, PTLD: Post-transplant Lymphoproliferative Disease, Scr: Serum Creatinine, LC: Light Chain, DM1: Diabetes Mellitus Type 1, MMF: Mycophenolate Mofetil.

	Hussain and Sureshkumar [5]	Khan et al. [6]	Tsuji et al. [7]	Crane et al. [8]	Albawardi et al. [9] (Two patients)
Native Kidney Disease	DM1 SPK TX	? CIN	FSGS	-	(1) DM type 1, (2) PKD
Age/Sex	38/F	59/F	41/M	18/M	(1) 24/M, (2) 68/F
Months/yrs Post Tx	6 yrs	19 yrs	4 yrs	3 yrs	(1) 3 yrs, (2) 13 yrs
Clinical Presentation	Raising Scr, Nephrotic proteinuria	Raising Scr, Microhematuria, 24-hour urine protein 2.4G	Protocol biopsy - mild proteinuria 0.3g/g creat	Mild proteinuria stable graft	Proteinuria and rising Scr
Biopsy Findings	Endocapillary hypercellularity, IGG3+ KappaLC+, C1Q+, C3+	Endocapillary proliferation, partial cellular crescents. IF- IGG3 and kappa +	Mesangial proliferation,IF-IGG, C1q, c3 Kappa LC +	Mesangial + MPGN IF – IGG+, kappa LC+	MPGN Monoclonal IGG+ and kappa light chain
Monoclonal Protein	Negative	Negative	Negative	Negative	Negative
Marrow plasmacytosis	Not reported	Negative	Negative	Not reported	Not reported
Treatment Given	Conservative-lisinopril	Chemotherapy	MMF held, rituximab	Conservative	Conservative
Response	Improving Scr and proteinuria in 18 months	Progressed to CKD5 14months	Improvement in proteinuria in 2 months	Stable	Not reported individually
Other Findings (viral infections, CLL, PTLD)	None	None	High copy EBV, PTLD suspected	None	None

The plausible pathogenic mechanism for de novo PGNMID is B cell activation with production of monoclonal immunoglobulins, most commonly IgG3κ, with subsequent glomerular deposition, complement activation, and renal injury [2,4]. This process is likely due to a small B cell or plasma cell clone that is undetectable and may be triggered by infections (such as parvovirus B19, hepatitis C, EBV) or hematological malignancy [4,5]. EBV infections may also accompany PTLD and subsequent PGNMID [7]. In this case, we excluded all possible infectious triggers. 

Clinical scenarios that mimic PGNMID include transplant glomerulopathy, Type 1 cryoglobulinaemic glomerulonephritis, primary immune complex membranoproliferative glomerulonephritis, or post-infectious glomerulonephritis [5]. Transplant glomerulopathy with concurrent immune deposits presents with polyclonal deposits of immunoglobulins. Since our patient had monoclonal kappa light chain positivity and negative immunostaining for lambda light chains, and negative staining for C4d, we excluded transplant glomerulopathy. 

Treatment options for PGNMID depend on the ability to identify monoclonal protein in the serum or urine and to detect a plasma or B-cell clone on bone marrow biopsy [1]. Looking for and managing infectious triggers is also important in de novo PGNMID. Clinical outcomes in PGNMID improve with clone-directed treatment [1,10]. For plasma cell-directed therapy, the usual approach to treatment is with bortezomib, cyclophosphamide, and dexamethasone [1]. Clinicians have also used daratumumab. For B cell-directed therapy, rituximab is used. However, if the clone is not detected, as is the case in most de novo PGNMID, empirical treatment of a hypothetical clone is considered after ruling out infectious triggers [1]. In patients with normal renal function, minimal proteinuria, and no detectable clone, researchers have used conservative treatment with angiotensin-converting enzyme inhibitor/angiotensin receptor blocker (ACEI/ARB). As our patient had significant renal dysfunction with proteinuria, we proceeded with empirical clone-directed therapy. Given the absence of a plasma cell clone and negative marrow findings, a B cell clone was hypothesized, prompting rituximab. We acknowledge that no post-treatment biopsy was done to confirm histological response. Management of de novo PGNMID shows variable therapeutic responses; some cases remain indolent or stabilize with conservative or immunosuppressive strategies [3]. Our patient’s allograft function stabilized after rituximab, consistent with occasional prior reports [3,10].

## Conclusions

De novo PGNMID is a rare clinical entity and presents much later than recurrent PGNMID. Identification of the clone responsible for PGNMID is difficult despite extensive evaluation in most patients. After ruling out infectious triggers for de novo PGNMID, such as EBV, HCV, and Parvovirus B19, and other non-plasma cell disorders such as low-grade lymphomas, CLL, and PTLD, we can give empirical clone-directed therapy if there is significant proteinuria and allograft dysfunction.
